# *RET* Lys666Asn has a low rate of MEN2-related tumors but may be associated with pheochromocytoma

**DOI:** 10.1007/s42000-026-00759-2

**Published:** 2026-03-19

**Authors:** Reut Halperin, Naama Peshes-Yaloz, Amit Tirosh, Orit Twito

**Affiliations:** 1 Division of Endocrinology, Metabolism, and Diabetes, Endocrine Neoplasia Translational Research Center, Sheba Medical Center, Tel Hashomer, Ramat Gan, Israel; 2 Gray Faculty of Medical and Health Sciences, Tel Aviv University, Tel Aviv, Israel; 3 Endocrinology and Diabetes Unit, Edith Wolfson Medical Center, Halochamim 62, Holon, Israel

**Keywords:** Multiple endocrine neoplasia 2, MEN2, Pheochromocytoma, Medullary thyroid carcinoma, MTC, Hyperparathyroidism

## Abstract

**Purpose:**

Multiple endocrine neoplasia type 2 (MEN2) is caused by germline pathogenic variants (PVs) in the *RET* proto-oncogene, leading to medullary thyroid carcinoma (MTC), pheochromocytoma, and primary hyperparathyroidism (PHPT). *RET* p.Lys666Asn is a rare PV, but its clinical significance remains incompletely understood. The purpose of this study is to expand the clinical understanding of the *RET* Lys666Asn variant.

**Methods:**

Index patients carrying the *RET* Lys666Asn variant were identified through clinical genetic testing in two referral medical centers. Comprehensive clinical evaluation, biochemical screening, and imaging studies were performed to assess the presence of MTC, pheochromocytoma, and PHPT of the index patients and carrier family members. We performed a literature search on Lys666Asn carriers and their clinical manifestations.

**Results:**

Ten individuals from five families were identified as carriers of the *RET* Lys666Asn variant. The median age at diagnosis was 43.7 years (range 2–75 years). Only one individual presented with pheochromocytoma, diagnosed at age 54. The rate of MTC, pheochromocytoma, and PHPT was 0 (0–0.26), 0.1 (0.01–0.39), and 0 (0–0.26), respectively. In a pooled analysis of cases reported in the literature, MTC, pheochromocytoma, and PHPT rate are 0.42 (0.28–0.57), 0.11 (0.04–0.22), and 0.03 (0–0.12), respectively. MTC had a wide age range at presentation (20–70 years).

**Conclusion:**

The *RET* Lys666Asn variant is associated with lower event rate of MEN2-related tumors compared to classical MEN2 variants, particularly MTC and pheochromocytoma. Despite its rarity, pheochromocytoma cannot be excluded in carriers, underscoring the need for individualized screening protocols based on specific *RET* variants.

**Supplementary Information:**

The online version contains supplementary material available at https://doi.org/10.1007/s42000-026-00759-2.

## Introduction

*RET* proto-oncogene gain of function alterations are the cause of multiple endocrine neoplasia type 2 (MEN2) syndrome. MEN2 is an autosomal dominant disorder including medullary thyroid carcinoma (MTC) in almost 100% of patients, pheochromocytoma in 50% of patients, and primary hyperparathyroidism (PHPT) in up to 35% of patients, divided into MEN2A and MEN2B [1]. MEN2B is a subgroup of MEN2, associated with MTC and pheochromocytoma, but does not include PHPT and often presents with marfanoid habitus and mucosal neuromas [2].

RET, a receptor tyrosine kinase, plays a crucial role in cellular processes such as proliferation, differentiation, and survival. Specific pathogenic variants (PVs) within the *RET* gene lead to a constitutively active receptor, driving the development of MTC and other associated neoplasms [3]. The strong genotype–phenotype correlation observed in MEN2 allows for targeted screening and personalized management of at-risk individuals, based on the classification of specific *RET* variants as moderate, high, or highest aggressive MTC risk integrated with the carrier’s age and marker levels [2, 4–6]. Indeed, this approach led to both a reduction of metastatic MTC and to an increase of biochemical cure after thyroidectomy, shifting the focus of MEN2 syndrome management to preventive medicine[7]. Management based on this classification method was recently challenged by a study demonstrating similar outcomes of the moderate and high-risk groups (however, the age of surgical intervention was markedly different) [8], and another study suggesting active surveillance in specific situations in order to prevent surgical intervention in early childhood[9].

The *RET* Lys666Asn variant (c.1998G > C, p.Lys666Asn) located on exon 11 is a rare MEN2a PV with a handful of reports in the literature. It is located near the transmembrane domain of the RET protein, causing increased kinase activity and possibly altering protein structure [10]. Prevalence is probably quite low. A recent literature review of MEN2 cases in the Mediterranean region did not report a PV in the 666 location [11], nor did a multicenter study of 275 patients with MTC in Israel [12]. MEN2-related event rate is also relatively low; the largest published cohort of *RET* Lys666Asn PV described C-cell abnormalities in 11 out of 24 carriers. The earliest age of MTC and C-cell hyperplasia in that cohort was 22 years and 20 years, respectively[13]. Several other reports of patients with MTC and Lys666Asn PV did not describe other MEN2-related manifestations [10, 14]. Pheochromocytoma in *RET* Lys666Asn variant carriers is probably extremely rare and was reported in two patients with apparently sporadic tumors[15] and in a single patient homozygous for the PV [16]. There are several other PVs located at amino acid 666; of note is Lys666Glu with reported cases of both MTC and pheochromocytoma [13, 17, 18].

Given the limited number of cases in the literature, the clinical spectrum and penetrance of the *RET* Lys666Asn variant remain poorly understood. This study aims to expand the clinical understanding of this variant by reporting five new, unrelated families carrying the *RET* Lys666Asn PV, including one family presenting with pheochromocytoma, an extremely rare manifestation of the *RET* Lys666Asn PV.

## Methods

### Study population

Patients were identified through clinical genetic testing at Sheba Medical Center Neuroendocrine Oncology Unit or Wolfson Medical Center Endocrinology Unit, Israel. A total of five unrelated index patients and their family members, all of diverse Jewish ancestry (Kindreds A-E, Table 1), were included in this study.

**Table 1:** Clinical data of ten *RET* c.1998G > C p.Lys666Asn germline PV carriers from five kindreds

Kindred	Patient no	Age at last follow- up (years)	Sex	Indications for genetic testing	Family history	MTC diagnosis Y/N	Tests/Age (years)	Pheo diagnosis Y/N	Tests/Age (years)	PHPT diagnosis Y/N	Tests/Age (years)	Other medical history
A	1 (index)	30	F	Perinatal evaluation	no	N	Cacitonin, CEA, US/30	N	Urine MN & NMN/30	N	Calcium/30	No
B	2 (index)	43	M	Perinatal evaluation	no	I	US thyroid nodules, largest 12 mm/42	NE	NE	NE	NE	No
	3	75	M	Family screening	no	I	CacitoninX4 ULN,US thyroid nodules largest 25 mm/75	NE	NE	NE	NE	MNG
C	4 (index)	37	F	Breast cancer	no	N	Calcitonin, US thyroid cyst 3 mm/37	NE	NE	N	Calcium/37	Breast cancer
	5	68	F	Family screening	no	N	Calcitonin, US thyroid/68	N	Urine MN & NMN/68	N	Calcium/68	Nephrolithiasis
D	6 (index)	36	F	Recurrent abortions	no	N	Calcitonin, US/36	N	Urine MN & NMN/36	N	Calcium/36	No
	7	66	M	Family screening	no	N	Thyroidectomy/66	I	Urine & NMN borderline, Abdominal CT no findings/66	N	Calcium/66	PTC
E	8 (index)	60	F	Pheochromocytoma	no	N	Calcitonin/60; Thyroidectomy/59	Y	Adrenalectomy/54	N	Calcium/60	MNG
	9	32	M	Family screening	yes	N	Plasma Calcitonin, US thyroid/32	N	Urine MN & NMN/32	N	Calcium/32	Crohn’s dis
	10	2	F	Family screening	yes	NE	NE	NE	NE	NE	NE	No

Calcitonin evaluated in plasma, calcium evaluated in serum. I -inconclusive results, N—no clinical and biochemical evidence of manifestation, NE – not evaluated. MNG – multinodular goiter; MTC – medullary thyroid cancer; Pheo – pheochromocytoma; PHPT – primary hyperparathyroidism; PTC – papillary thyroid cancer; PV – pathogenic variant

Clinical evaluation.

All identified carriers underwent a comprehensive clinical evaluation, including biochemical screening for MTC (plasma calcitonin levels), pheochromocytoma (urinary fractionated metanephrine and normetanepheine levels), and PHPT (serum calcium and parathyroid hormone levels). Imaging studies were performed as indicated to assess the presence of MTC, pheochromocytoma, or other related neoplasms.

### Screening of family members

All first-degree family members were requested to complete genetic screening for *RET* Lys666Asn PV. Family members from kindreds A-C were assessed in Sheba MC using Sanger sequencing (detailed below) after providing informed consent. Family members from kindreds D and E were assessed in validated genetic laboratories (ethical approval numbers SMC-18–5674 & WMC-0143–21).

### Genetic evaluation

**Fig. 1:**
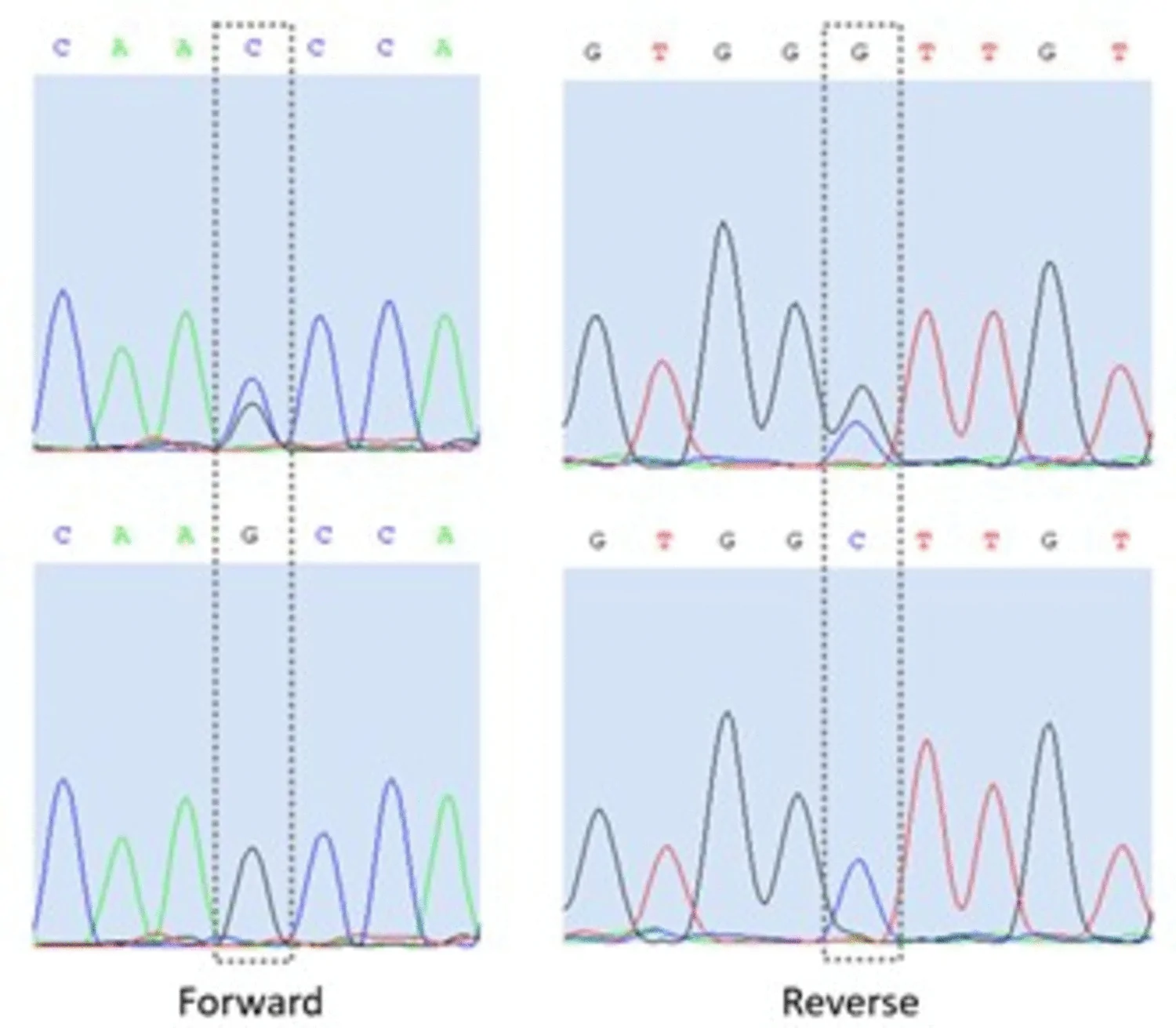
Sanger sequencing of the *RET* c.1998G > C p.Lys666Asn pathogenic variant (PV). Germline DNA of PV carrier and non-carrier (upper and lower rows, respectively)

The DNA from peripheral blood mononuclear cells was purified using the Quick-DNA/RNA Microprep Plus Kit (Zymo Research, Freiburg, Germany). PCR amplification of *RET* exon 11 was performed using the EZ-3007 PCR kit (Hylabs), with the forward primer CCTATGCTTGCGACACCAGT and the reverse primer CCTCCCAGCAATTTCCTCCC. The resulting PCR products were sequenced and analyzed using the SangerseqR package on the RStudio platform (version 2022.02.0, PBC) [14]. The resulting sequences were compared to PV positive family members and healthy unrelated controls. Figure 1 shows the *RET* gene codon 666 locus, with an example of a wildtype allele, and of a heterozygous *RET* c.1998G > C p.Lys666Asn.

### Literature review and pooled analysis

We performed a thorough literature search on the PUMBED database for publications in the English language reporting patients with an alteration in amino acid *666* of the RET protein. We used the following search terms: 1. (((MEN2) OR (multiple endocrine neoplasia type 2) OR (RET)) and 666), resulting in 11 results; and 2. (((MEN2) OR (multiple endocrine neoplasia type 2)) and cohort) resulting in 106 records. All papers were manually screened for reports including patients with a variant in the 1998 codon of the *RET* gene or a variant in the 666 amino acid in the RET protein, and for references to additional reports including such patients (Supplementary Fig. 1). Duplications were omitted.

### Statistical analysis

For the local and pooled cohort, exact Clopper-Pearson 95% CIs were calculated using the GenBinomApps R package. A random-effects meta-analysis was conducted on familial event proportions using the metaprop function of the meta R package with a logit transformation, Wilson score confidence intervals, and a continuity correction to account for studies with zero events, assessing heterogeneity with ${I}^{2}$ and ${\tau }^{2}$. The Kaplan–Meier survival analysis was performed using the survival and survminer packages in R. Event indicators for MTC, pheochromocytoma, and PHPT were coded as binary variables, and age at event or last follow-up was used as the time variable. Survival objects were created with the Surv() function, followed by Kaplan–Meier curve estimation with survfit(). The ggsurvplot() function was used to generate survival plots with confidence intervals.

## Results

**Fig. 2:**
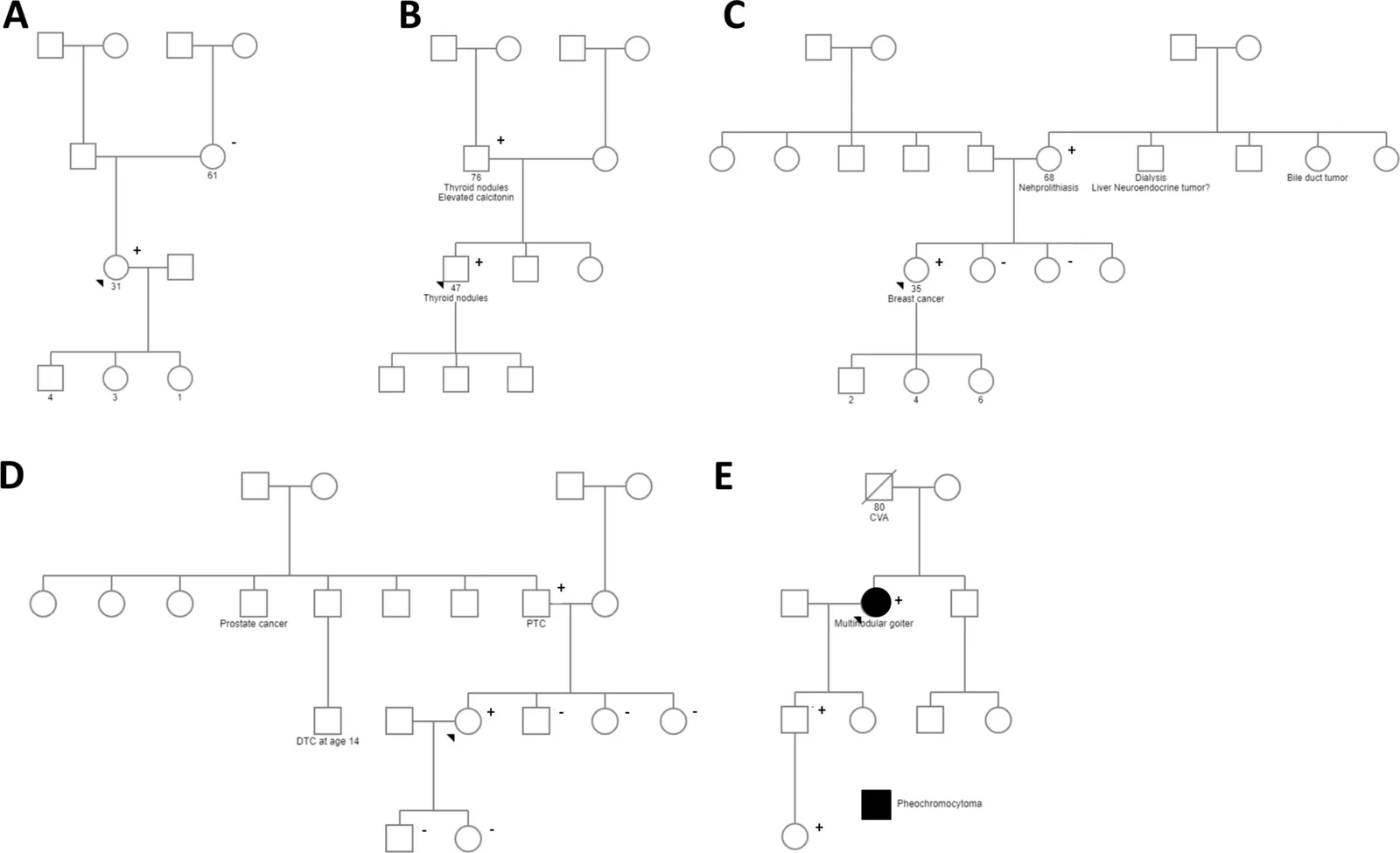
Pedigrees of five kindreds (**A**-**E**) with a familial germline *RET* c.1998G > C p.Lys666Asn pathogenic variant (PV), showing low penetrance, but a presentation with pheochromocytoma. Plus symbol (+) designates a PV carrier, minus symbol (-) designates a non-PV carrier. Numbers indicate current age in years. CVA – cerebrovascular accident; DTC – differentiated thyroid cancer; PTC – papillary thyroid cancer.

A total of 10 (out of 18 evaluated) individuals from five unrelated families of Jewish ancestry were identified as carriers of the *RET* c.1998G > C p.Lys666Asn PV. Only one patient was evaluated due to MEN2-related clinical diagnosis (proband in kindred E) with subsequent evaluation of next-of kins. The average age at diagnosis was 43.7 years (median 40; range 2–75 years). The clinical characteristics of these individuals are summarized in Table 1, and their pedigrees are presented in Fig. 2. There was no significant difference in the rate of clinical manifestations between index patients and their relatives (Supplementary Table 1).

Kindred A: The index patient, a 30-year-old woman, underwent exome sequencing during pregnancy due to fetal abnormalities and was found to carry the *RET* Lys666Asn PV. Her calcitonin, carcinoembryonic antigen (CEA), 24-h urine metanephrines, and calcium levels were all normal. Her family history was negative for MEN2 manifestations, although only her mother underwent genetic testing and was negative.

Kindred B: The index patient, a 43-year-old male, and his fetus were found to harbor the Lys666Asn PV during trio exome sequencing for pregnancy evaluation. He had non-suspicious thyroid nodules with normal calcitonin levels and was normocalcemic. Although asymptomatic for pheochromocytoma, he did not perform metanephrine testing. His 75-year-old father, also a carrier, was formerly diagnosed with benign multinodular goiter and had elevated serum calcitonin (48.7 ng/l, reference range 0–13) but did not undergo further evaluation.

Kindred C: The index patient, a 35-year-old woman, was diagnosed with breast cancer and tested positive for the *RET* Lys666Asn PV. Repeated FDG-PET/CT scans showed no thyroid or adrenal lesions and her CEA and calcium levels were normal. Plasma calcitonin and urinary fractionated metanephrine levels were not tested. Her 58-year-old mother, also a carrier, had normal thyroid sonography, plasma calcitonin, and urinary fractionated metanephrine levels.

Kindred D: The index patient, a 36-year-old woman, underwent genetic testing after recurrent miscarriages and her daughter’s leukemia diagnosis at the age of 2 months. She carried the Lys666Asn PV and additional variants of uncertain significance (*RET* Arg883His, and in *CDH1*, *HOXB13*, and *MECOM*). At 36, she had non-suspicious thyroid nodules, normal plasma calcitonin, calcium, and normal urinary fractionated metanephrine levels. Her father, who had papillary thyroid cancer, was the source of the variant but had no MEN2A symptoms at the age of 66 years. Her paternal grandfather died at the age of 90 years and her grandmother at the age of 74 years: neither had any MEN2-related disease.

Kindred E: The 60-year-old index patient was diagnosed with pheochromocytoma at age 54, due to symptoms of recurrent syncope and hypertension, and was found to carry the *RET* Lys666Asn PV. She underwent laparoscopic left adrenalectomy resection with a finding of a 3 cm pheochromocytoma. During 6-years follow-up, there was no evidence of recurrence. A few years earlier, she underwent total thyroidectomy for multinodular goiter with benign pathology. Post-thyroidectomy calcitonin levels were undetectable. Her 32-year-old son, also a carrier, had a normal thyroid ultrasound and calcium levels, with no symptoms of pheochromocytoma. His 2-year-old daughter, a carrier, was referred for pediatric endocrinology evaluation.

### Literature review

In addition to the cohort described above, we identified a total of 38 reported cases of individuals carrying the *RET* p.Lys666Asn PV: some had c.1998G > C and some c.1998G > T, although the specific variant was not reported for all cases (Table 2) [10, 13, 16, 19–21]. Most of these individuals were evaluated due to a personal or family history of MTC. Of the 38 carriers, 15 (39.4%) were diagnosed with MTC, with a median age of diagnosis of 49 years [range 20–70]. Only six patients had MTC that was not confined to the thyroid: four (26.7%) had cervical lymph node involvement and two (13.3%) had distant metastases. Of note, the youngest age for lymph node involvement or distant metastases were 22 years of age and 23 years of age, respectively. Four carriers (10.8%) had pheochromocytoma, including one who was homozygous for the PV. One patient had hyperparathyroidism.

**Table 2:** Previously published cases of *RET* p.Lys666Asn germline PV carriers. Numbers in parentheses indicate age at diagnosis, and for MTC indicate age at thyroidectomy

Reference	Variant	Kindred	Patient no	Age at last follow-up (years)	Sex	Indications for genetic testing	Relevant family history	MTC (age^)	Pheo	PHPT	Notes
Muzza et al. 2010 (Italy)	c.1998G > T	A	1 (index)	65	F	MTC	No	Y (65)	N	N	
Xu- et al. 2016 (Texas, USA)	Not stated	B	2 (index)	55	F	MTC	No	Y (22)	N	N	LN involvement
			3	20	F	Family screening	Yes	Y (20)	N	N	CCH
											Daughter of #2
		C	4 (index)	34	F	MTC	No	Y (33)	N	N	Distant mets
			5	57	F	Family screening	Yes	*	N	N	Mother of #4
			6	80	F	Family screening	Yes	N	NE	NE	Grandmother of #4
		D	7 (index)	32	F	MTC	No	Y (23)	N	N	Distant mets
			8	58	M	Family screening	Yes	N	NE	NE	Father of #7
			9	27	F	Family screening	Yes	N	NE	NE	Sister of #7
		E	10 (index)	65	F	MTC	Yes	Y (49)	N	N	LN involvement, sister had PHPT
		F	11 (index)	59	M	MTC	No	Y (51)	N	N	Also had PTC
		G	12 (index)	61	F	MTC	No	Y (59)	N	N	LN involvement
			13	30	F	Family screening	Yes	N (30)	NE	NE	Daughter of #12
			14	41	F	Family screening	Yes	N (30)	NE	NE	Daughter of #15
			15	58	M	Family screening	Yes	NE	NE	NE	Brother of #12
			16	47	F	Family screening	Yes	NE	NE	NE	Sister of #12
			17	5	M	Family screening	Yes	NE	NE	NE	Son of # 14
		H	18 (index)	65	F	MTC	No	Y (64)	N	N	
			19	20	M	Family screening	Yes	N (20)	N	N	Son of # 18
		I	20 (index)	64	F	MTC	No	Y (55)	N	N	
			21	92	F	Family screening	Yes	NE	NE	NE	Mother of # 20
			22	70	F	Family screening	Yes	Y (70)	N	N	Sister of # 20
			23	39	F	Family screening	Yes	N	NE	NE	Daughter of # 22
			24	61	M	Family screening	Yes	Inconclusive	NE	NE	Brother of # 20
			25	29	M	Family screening	Yes	NE	NE	NE	Son of # 24
Jaber et al. 2018 (Texas, USA)	Not stated	J	26 (index)	59	F	MTC	Yes	Y (59)	Y(59)	N	Homozygous for Lys666Asn,
											LN involvement, Bilateral Pheo
			27	32	M	Family screening	Yes	Y (32)	N	N	Son of #26
			28	30	F	Family screening	Yes	CCH (30)	N	N	Daughter of #26
			29	61	F	Family screening	Yes	N	N	N	Sister of #26
			30	43	F	Family screening	Yes	N	N	N	Daughter of #29
			31	25	M	Family screening	Yes	N	NE	NE	Son of #29
Curras-Freixes et al. 2015 (Spain)	c.1998G > C	K	32 (index)	32	M	Pheo	No	N	Y (32)	N	
Yehia et al. 2018 (Ohio, USA)	c.1998G > T	L	33 (index)	58	M	Suspected Cowden syndrome	No	N	Y (49)	N	Renal cancer, differentiated thyroid cancer
La Greca et al. 2024 (Colorado, USA)	c.1998G > T	M	34 (index)	40	F	Pheo	No	Y (36)	Y (36)	Y (36)	Presented with a perioperative hypertensive crisis. MTC was single microcarcinoma with LN involvement
			35	42	F	Family screening	Yes	N	N	N	Sister of #34, metastatic PTC
			36	46	M	Family screening	Yes	Inconclusive	N	N	Brother of #34
			37	22	F	Family screening	Yes	N	N	N	Daughter of #34
			38	NA	NA	Family screening	Yes	NE	NE	NE	Child of #35

^Age at thyroidectomy; *—elevated calcitonin and ultrasound finding, refused surgery; CCH – C-cell hyperplasia; LN – MTC lymph node involvement; mets. – MTC metastases; MTC – medullary thyroid carcinoma; N – no, Pheo – pheochromocytoma; PHPT – primary hyperparathyroidism; PTC – papillary thyroid carcinoma; PV – pathogenic variant; Y—yes. N—no clinical and biochemical evidence of manifestation, NE – not evaluated

**Fig. 3:**
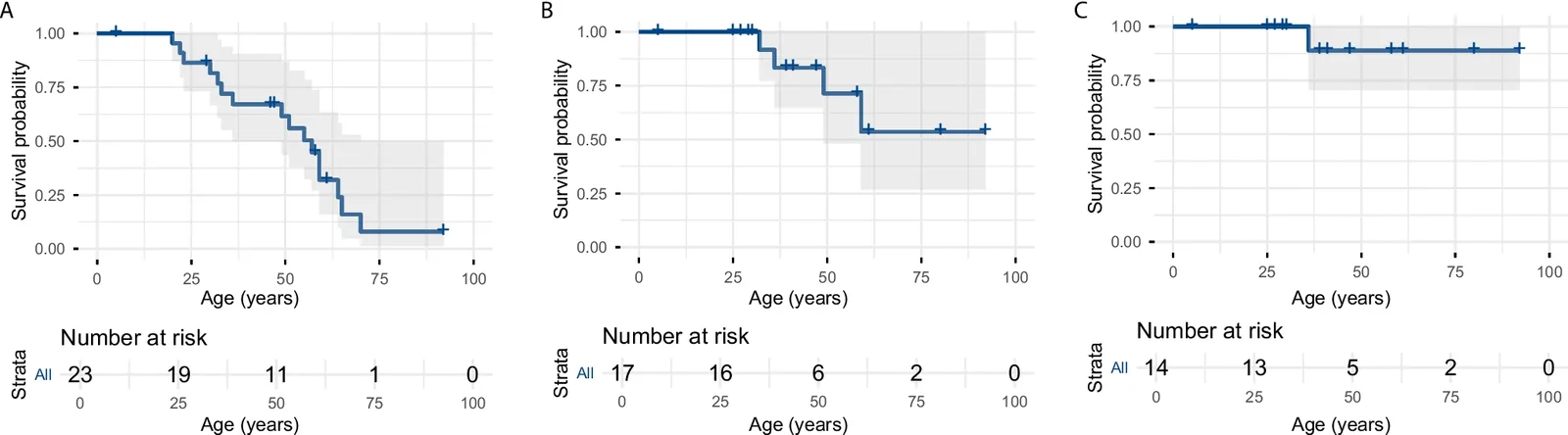
Kaplan–Meier curves with Greenwood SE bands for risk of MTC (left), pheochromocytoma (middle), and PHPT (right) for the pooled cohort

The pooled proportions for MTC, pheochromocytoma, and primary hyperparathyroidism (PHPT) across families (random-effects meta-analysis of event proportions) are approximately 63%, 47%, and 33%, respectively, with minimal heterogeneity (${I}^{2}$ near 0%) indicating consistent effects across families, though heterogeneity estimates (${\tau }^{2}$, etc.) suggest some variability, especially for PHPT (Supplementary Fig. 2). Detailed data of these patients are presented in Table 2. Kaplan–Meier plots of MEN2-related manifestations in the pooled literature cohort are presented in Fig. 3, demonstrating that most MTC events occurred past the age of 50, with a low rate of pheochromocytoma and PHPT. Age distribution of MTC and pheochromocytoma for the pooled locale and literature cohorts combined is presented in Supplementary Fig. 3, demonstrating that MTC can occur from the age of 20 up to 70. There was no significant difference in the rate of clinical manifestations between index patients and their relatives (Supplementary Table 2).

Comparing the data from the current cohort and the literature review, the rate of MTC, pheochromocytoma, and PHPT in our cohort (95% CI) are 0 (0—0.26), 0.1 (0.01—0.39), and 0 (0—0.26), respectively, while their respective rates based on the literature are 0.42 (0.28—0.57), 0.11 (0.04—0.22), and 0.03 (0—0.12), respectively. Of note, only two (13.3%) patients had an aggressive disease involving distant metastases.

## Discussion

The *RET* c.1998G > C p.Lys666Asn (Lys666Asn) variant is a rare cause of MEN2, with limited data being available on its clinical implications. The current study adds to the existing literature by reporting 10 individuals from five new families carrying the *RET* Lys666Asn variant, including a very rare case of pheochromocytoma in a heterozygous carrier.

Although genotype–phenotype association is well established for most *RET* variants [4], data on the phenotype of Lys666Asn are scarce due to the extremely small number of cases described in the medical literature (Table 2). The c.1998G > C and c.1998G > T p.Lys666Asn variants are classified as pathogenic/likely pathogenic by the ClinVar National Institution of Health (NIH) database [22],with a frequency of 0.0000055 and 0.0000143 in gnomAD (version 4.1.0), respectively. Experimental studies have shown that the c.1998G > T variant results in increased RET phosphorylation activity and increased transformation potential in cell culture[10]. Codon 666 of the *RET* gene encodes evolutionary conserved residues in the intra-cellular juxta-membrane domain of RET. Other missense variants at the same codon (p.Lys666Glu, p.Lys666Met, p.Lys666Arg) including a c.1998delinsTTCT have been reported in individuals with MTC and MEN2 syndrome, supporting the importance of the lysine residue in that location for RET protein function [17, 18, 23].

Pathogenic variants in codon 666 in general are rare. A comprehensive review of the global spread of *RET* variants summarized data from the seven largest series of *RET* variants from Europe, North America, South America, and Japan. PVs in codon 666 were found in only 0.81% (20/2488) of cases[24]. The specific Lys666Asn PV is probably extremely rare, with only one case series and several case reports published currently; it was not detected in multiple cohorts of MTC in the Mediterranean region and, specifically, was not detected in an Israeli multicenter study of *RET* variants in MTC[11, 12]. The limited amount of data on this PV may represent its real prevalence, but may also be influenced by under-diagnosis, as tumors associated with Lys666Asn PV may mistakenly be considered as sporadic tumors due to older age tumor development and paucity of relevant family history.

Lys666Asn alteration was reported in 38 patients in the literature thus far, to the best of our knowledge, with a mixture of reports for c.1998G > T and c.1998G > C variants. Most patients were diagnosed due to a MEN2-related diagnosis or as part of family screening due to known MEN2 syndrome in the family. Overall, the 39.4% rate of MTC, with four cases involving lymph nodes and two cases of distant metastatic MTC (some presenting in their early twenties), four (10.5%) cases of pheochromocytoma, and one PHPT. The first report was a case of seemingly sporadic MTC in a 65-year-old female [10]. A few years later, Xu et al. reported data of eight probands with MTC, including data on 16 family members positive for this PV. In that cohort, 13/24 (54.2%) carriers had no evidence of MTC or C-cell hyperplasia in the age range of 21–80 years. The authors conclude that this alteration is associated with low MTC penetrance and phenotypic variability [13]. Jaber et al. published a case of a female homozygous for Lys666Asn who was diagnosed with MTC and bilateral pheochromocytoma. Five of her family members were found to be carriers of the variant, three of them with no C-cell pathology (3/5, 60%) at the age of 25–61 years [16]. Our series supports the lower-than-expected event rate of MTC and/or older age of presentation in *RET* c.1998G > C p.Lys666Asn PV carriers compared to classical MEN2 variants. In our cohort, eight of nine (8/9, 88.9%) adult subjects had no evidence of MTC or C-cell hyperplasia in the age range of 30–66 years. Only one subject (patient no. 3) had high plasma calcitonin level and a multinodular goiter. However, at the age of 75 years he did not have clinical evidence of overt MTC. Unfortunately, he dropped out of follow-up. No other subjects had clinical, radiologic, or laboratory evidence of MTC, and their family history was negative for MEN2-related manifestations. As in previous cohorts, none of our patients had PHPT.

The low MTC event rate in our cohort might result from a relatively low MTC risk of this specific variant, although definitive classification requires age-specific penetrance under standardized ascertainment. Another possible explanation could be that this cohort consisted of patients who underwent genetic evaluation due to non-MEN2-related diagnoses and extended follow-up could identify additional cases of MTC. That is, young unaffected carriers might still be at risk due to late onset, requiring continuous surveillance. In addition, disease event rate in MEN2 is probably dependent not only on the specific variant but also on other factors such as epigenetic changes[25]. For example, Loveday et al. [26], found a high carrier frequency of the moderate-risk RET PV Val804Met in patients who underwent genetic evaluation due to non-cancer related conditions, suggesting a lower penetrance rate compared to cohorts of patients evaluated due to family history of MTC.

In this study, one of the patients presented with a *RET* compound heterozygous state, however, with no clinical manifestations. A compound heterozygous state is rarely reported in the context of MEN2[27]. A single gain-of-function hit is necessary but may not be sufficient for MEN2 syndrome to develop[28, 29]: for example, several studies demonstrate that allelic imbalance also plays a role in tumor development in individuals with *RET* germline mutations[28, 30, 31]. This is in contrast to a loss of heterozygosity required in other syndromes (e.g., in MEN1 syndromes[32]). Two case reports describe a compound heterozygous state, one of a family of C634Y (a high-risk variant) and Val292Met (unknown significance) variants, with a slightly more aggressive phenotype in the two family members with the compound carrier state compared to others ([33]). Another report includes a family with a combination of Val804Leu (moderate risk) and Val648Ile (a variant with conflicting classification); the proband developed pheochromocytoma, while other carriers had no clinical MEN2-related diagnoses [34].

Pheochromocytoma was detected in a single patient in our cohort. The tumor was diagnosed at the age of 54 years, with classical signs and symptoms. During 6-years follow-up, there was no evidence of recurrence. A thorough literature review revealed only handful of cases of pheochromocytoma associated with *RET* Lys666Asn PV. Pheochromocytoma was identified in one patient from a screen of 329 patients with apparently sporadic paraganglioma or pheochromocytoma (a 32 year old with no other MEN2 manifestations or relevant family history [21], in a patient with Cowden’s syndrome (a 49 year old male who additionally suffered from differentiated thyroid cancer and renal cell cancer, with no report of MTC or PHPT evaluation [20]), in a patient homozygous to this PV (59 year old female with metastatic MTC and bilateral pheochromocytoma)[16] and in a 40 year old patient presenting with hypertensive crisis [19].

## Study limitations

The small sample size of our study limits the generalizability of the findings and the retrospective nature of the data collection may introduce bias. Future studies with larger cohorts are needed to confirm these findings and provide a more comprehensive understanding of the clinical course associated with the *RET* Lys666Asn variant. In addition, in this study we pooled literature data on carriers of two distinct variants (c.1998G > C and 1998G > T). While both result in the same protein sequence, splicing variation may occur. Furthermore, pooled comparisons performed in this analysis may be misleading due to intra-kindred correlation. To resolve this bias, we performed statistical correction, but this led to model overfitting, a limitation often encountered in rare disease with sparse events. Therefore, the comparisons presented should be interpreted cautiously. Additionally, functional studies exploring the molecular mechanisms of the *RET* Lys666Asn variant could offer insights into its pathogenicity and guide therapeutic strategies.

## Conclusion

*RET* p. Lys666Asn germline PV is a rare cause of MEN2 syndrome, with observed proportions to date, consistent with lower event rates than classical MEN2 variants and older age of MEN2-related tumor development. However, definitive variant risk classification requires age-specific penetrance under standardized ascertainment. The occurrence of pheochromocytoma in only five reported cases to date underscores the rarity of this manifestation among carriers of the *RET* Lys666Asn variant, emphasizing the need for individualized screening protocols tailored to the specific *RET* variant. This information is crucial for genetic counseling, as it provides a more personalized risk assessment for individuals carrying the *RET* Lys666Asn variant.

## Supplementary Information

Below is the link to the electronic supplementary material.

Supplementary file1 (DOCX 19 KB)

Supplementary file2 (DOCX 18 KB)

Supplementary file3 (PPTX 36 KB)

Supplementary file4 (PDF 231 KB)

Supplementary file5 (PDF 44 KB)

## Data Availability

All data are provided within this manuscript.
