# Supplementary material for: RET Lys666Asn has a low rate of MEN2-related tumors but may be associated with pheochromocytoma
Source: Hormones (Athens). 2026 Mar 19;25(2):365–75. doi: 10.1007/s42000-026-00759-2 (PMC13582063; doi:10.1007/s42000-026-00759-2)
Supplement: Supplementary file 1 [file 42000_2026_759_MOESM4_ESM.pdf]

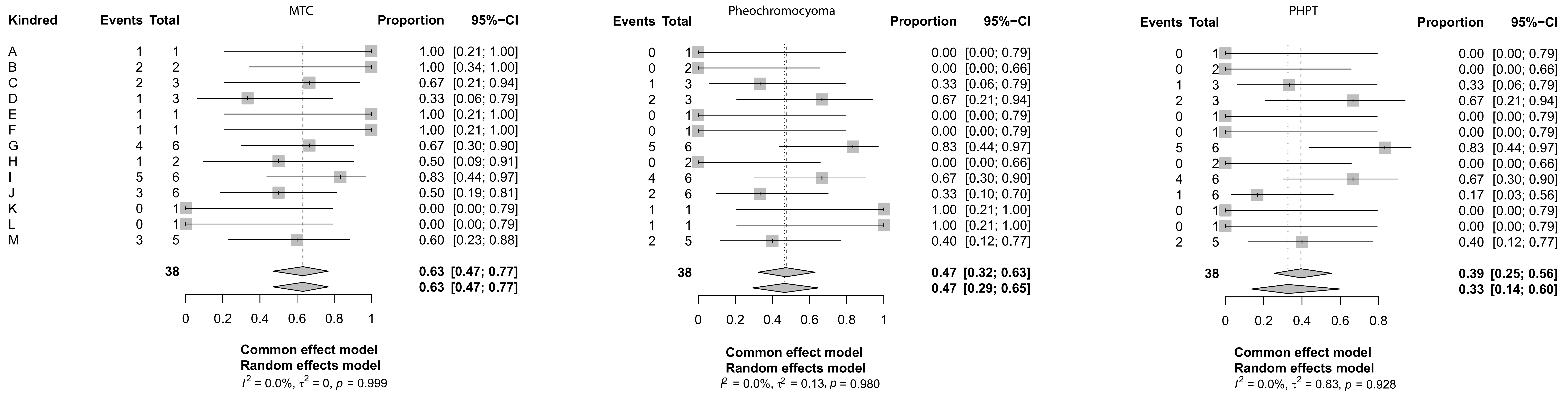
MTC
Kindred Events Total Proportion 95%−CI
A 1 1 1.00 [0.21; 1.00]
B 2 2 1.00 [0.34; 1.00]
C 2 3 0.67 [0.21; 0.94]
D 1 3 0.33 [0.06; 0.79]
E 1 1 1.00 [0.21; 1.00]
F 1 1 1.00 [0.21; 1.00]
G 4 6 0.67 [0.30; 0.90]
H 1 2 0.50 [0.09; 0.91]
I 5 6 0.83 [0.44; 0.97]
J 3 6 0.50 [0.19; 0.81]
K 0 1 0.00 [0.00; 0.79]
L 0 1 0.00 [0.00; 0.79]
M 3 5 0.60 [0.23; 0.88]
38 0.63 [0.47; 0.77]
0.63 [0.47; 0.77]
0 0.2 0.4 0.6 0.8 1
Common effect model
Random effects model
I^2 = 0.0%, τ^2 = 0, p = 0.999
Pheochromocytoma
Events Total Proportion 95%−CI
0 1 0.00 [0.00; 0.79]
0 2 0.00 [0.00; 0.66]
1 3 0.33 [0.06; 0.79]
2 3 0.67 [0.21; 0.94]
0 1 0.00 [0.00; 0.79]
0 1 0.00 [0.00; 0.79]
5 6 0.83 [0.44; 0.97]
0 2 0.00 [0.00; 0.66]
4 6 0.67 [0.30; 0.90]
2 6 0.33 [0.10; 0.70]
1 1 1.00 [0.21; 1.00]
1 1 1.00 [0.21; 1.00]
2 5 0.40 [0.12; 0.77]
38 0.47 [0.32; 0.63]
0.47 [0.29; 0.65]
0 0.2 0.4 0.6 0.8 1
Common effect model
Random effects model
I^2 = 0.0%, τ^2 = 0.13, p = 0.980
PHPT
Events Total Proportion 95%−CI
0 1 0.00 [0.00; 0.79]
0 2 0.00 [0.00; 0.66]
1 3 0.33 [0.06; 0.79]
2 3 0.67 [0.21; 0.94]
0 1 0.00 [0.00; 0.79]
0 1 0.00 [0.00; 0.79]
5 6 0.83 [0.44; 0.97]
0 2 0.00 [0.00; 0.66]
4 6 0.67 [0.30; 0.90]
1 6 0.17 [0.03; 0.56]
0 1 0.00 [0.00; 0.79]
0 1 0.00 [0.00; 0.79]
2 5 0.40 [0.12; 0.77]
38 0.39 [0.25; 0.56]
0.33 [0.14; 0.60]
0 0.2 0.4 0.6 0.8
Common effect model
Random effects model
I^2 = 0.0%, τ^2 = 0.83, p = 0.928
