# Supplementary material for: RET Lys666Asn has a low rate of MEN2-related tumors but may be associated with pheochromocytoma
Source: Hormones (Athens). 2026 Mar 19;25(2):365–75. doi: 10.1007/s42000-026-00759-2 (PMC13582063; doi:10.1007/s42000-026-00759-2)
Supplement: Supplementary file 2 [file 42000_2026_759_MOESM5_ESM.pdf]

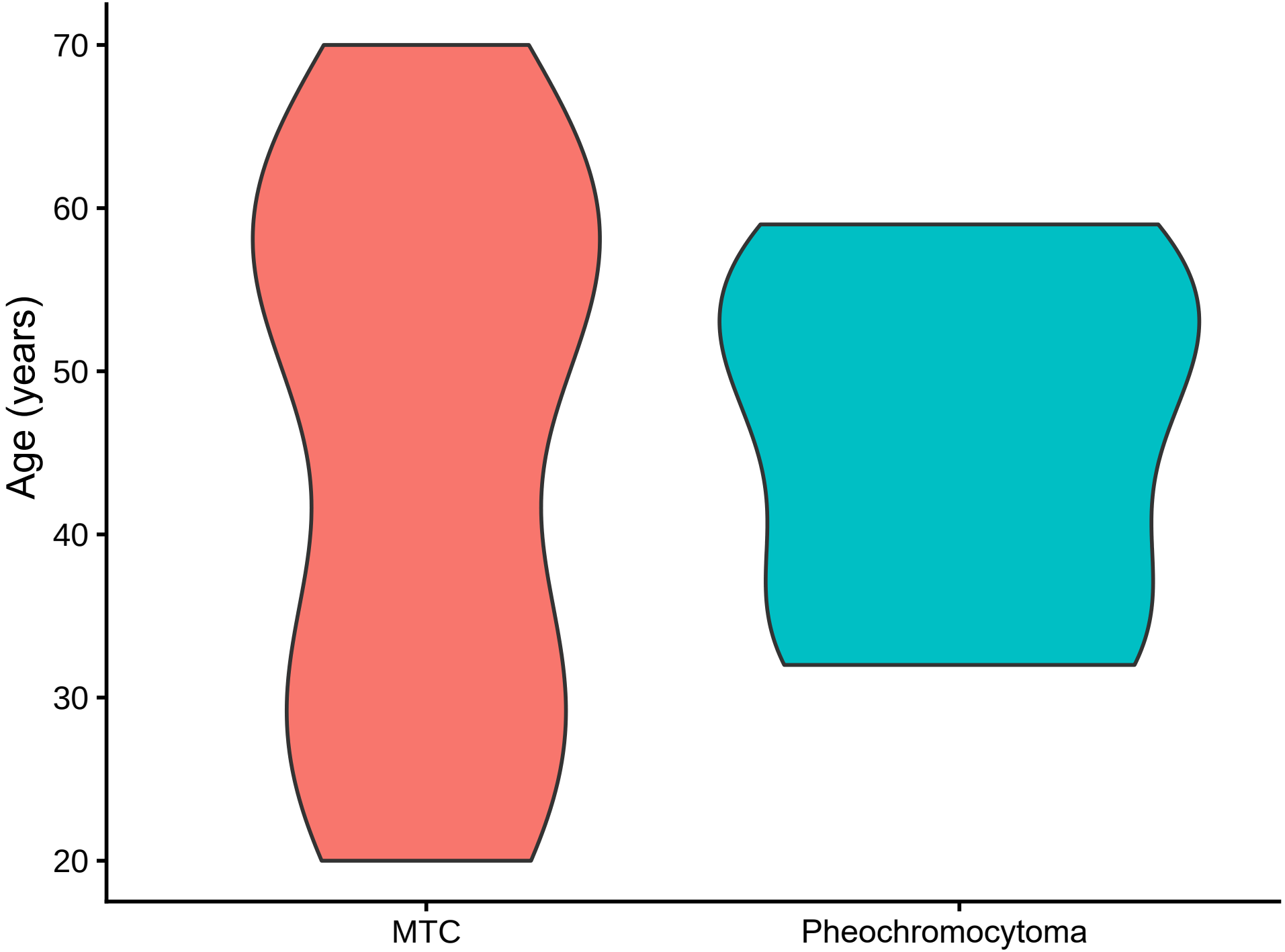

Age (years)
70
60
50
40
30
20
MTC
Pheochromocytoma
