# Supplementary material for: RET Lys666Asn has a low rate of MEN2-related tumors but may be associated with pheochromocytoma
Source: Hormones (Athens). 2026 Mar 19;25(2):365–75. doi: 10.1007/s42000-026-00759-2 (PMC13582063; doi:10.1007/s42000-026-00759-2)
Supplement: Supplementary file 3 [file 42000_2026_759_MOESM1_ESM.docx]

**Supplementary Table 1.** Comparison of clinical manifestations between the index patients and their relatives in the local cohort.

|  | Overall | Index | Relative | p value |
| --- | --- | --- | --- | --- |
| N | 10 | 5 | 5 |  |
| Kindred (%) |  |  |  | 0.856 |
| A | 1 (10.0) | 1 (20.0) | 0 ( 0.0) |  |
| B | 2 (20.0) | 1 (20.0) | 1 ( 20.0) | |
| C | 2 (20.0) | 1 (20.0) | 1 ( 20.0) | |
| D | 2 (20.0) | 1 (20.0) | 1 ( 20.0) | |
| E | 3 (30.0) | 1 (20.0) | 2 ( 40.0) | |
| Patient no. (mean (SD)) | 5.50 (3.03) | 4.20 (2.86) | 6.80 (2.86) | 0.189 |
| Age at last follow-up years (mean (SD)) | 44.90 (22.33) | 41.20 (11.48) | 48.60 (30.92) | 0.629 |
| Sex Male n(%) | 4 (40.0) | 1 (20.0) | 3 ( 60.0) | 0.519 |
| Indications for genetic testing (%) |  |  |  | 0.04 |
| Breast cancer | 1 (10.0) | 1 (20.0) | 0 ( 0.0) |  |
| Family screening | 5 (50.0) | 0 ( 0.0) | 5 (100.0) | |
| Perinatal evaluation | 2 (20.0) | 2 (40.0) | 0 ( 0.0) |  |
| Pheochromocytoma | 1 (10.0) | 1 (20.0) | 0 ( 0.0) |  |
| Recurrent abortions | 1 (10.0) | 1 (20.0) | 0 ( 0.0) |  |
| Relevant family history Yes n(%) | 2 (20.0) | 0 ( 0.0) | 2 ( 40.0) | 0.429 |
| MTC diagnosis No diagnosis n(%) | 7 (70.0) | 4 (80.0) | 3 ( 60.0) | 1 |
| Pheo diagnosis |  |  |  | 0.549 |
| Evaluated n(%) | 5 (50.0) | 2 (40.0) | 3 ( 60.0) | |
| Pheo diagnosis Yes n(%) | 1 (10.0) | 1 (20.0) | 0 ( 0.0) |  |
| No pheo diagnosis n(%) | 4 (40.0) | 2 (40.0) | 2 ( 40.0) | |
| PHPT diagnosis No n(%) | 7 (70.0) | 4 (80.0) | 3 ( 60.0) | 1 |
| Other medical history (%) |  |  |  | 0.416 |
| Crohn’s disease | 1 (10.0) | 0 ( 0.0) | 1 ( 20.0) | |
| Breast cancer | 1 (10.0) | 1 (20.0) | 0 ( 0.0) |  |
| MNG | 2 (20.0) | 1 (20.0) | 1 ( 20.0) | |
| Nephrolithiasis | 1 (10.0) | 0 ( 0.0) | 1 ( 20.0) | |
| No | 4 (40.0) | 3 (60.0) | 1 ( 20.0) | |
| PTC | 1 (10.0) | 0 ( 0.0) | 1 ( 20.0) | |
