# Supplementary material for: RET Lys666Asn has a low rate of MEN2-related tumors but may be associated with pheochromocytoma
Source: Hormones (Athens). 2026 Mar 19;25(2):365–75. doi: 10.1007/s42000-026-00759-2 (PMC13582063; doi:10.1007/s42000-026-00759-2)
Supplement: Supplementary file 4 [file 42000_2026_759_MOESM2_ESM.docx]

**Supplementary Table 2.** Comparison of clinical manifestations between the index patients and their relatives in the literature-pooled cohort.

|  | Overall | Index | Relatives | p |
| --- | --- | --- | --- | --- |
| N | 38 | 13 | 25 |  |
| Variant (%) |  |  |  | 0.302 |
| c.1998G>C | 1 (2.6) | 1 (7.7) | 0 (0.0) |  |
| c.1998G>T | 7 (18.4) | 3 (23.1) | 4 (16.0) | |
| Not stated | 30 (78.9) | 9 (69.2) | 21 (84.0) | |
| Relatives n(%) | 25 (65.8) | 0 (0.0) | 25 (100.0) | <0.001 |
| Age of last follow-up years (mean (SD)) | 46.59 (18.98) | 53.00 (13.32) | 43.12 (20.87) | 0.133 |
| Sex = M (%) | 12 (32.4) | 3 (23.1) | 9 (37.5) | 0.598 |
| Indications for genetic testing n(%) |  |  |  | <0.001 |
| Family screening | 25 (65.8) | 0 (0.0) | 25 (100.0) | |
| MTC | 10 (26.3) | 10 (76.9) | 0 (0.0) |  |
| Pheo | 2 (5.3) | 2 (15.4) | 0 (0.0) |  |
| Suspected Cowden syndrome | 1 (2.6) | 1 (7.7) | 0 (0.0) |  |
| Relevant family history Yes n(%) | 27 (71.1) | 2 (15.4) | 25 (100.0) | <0.001 |
| MTC diagnosis age (mean (SD)) | 44.35 (18.98) | 46.62 (15.40) | 43.12 (20.87) | 0.6 |
| Pheo diagnosis age (mean (SD)) | 46.24 (18.94) | 52.00 (13.63) | 43.12 (20.87) | 0.177 |
| PHPT diagnosis age (mean (SD)) | 46.49 (19.03) | 52.69 (13.69) | 43.12 (20.87) | 0.147 |
| MTC diagnosis_Yes n(%) | 16 (66.7) | 11 (100.0) | 5 (38.5) | 0.006 |
| Pheo_diagnosis Yes n(%) | 4 (22.2) | 4 (100.0) | 0 (0.0) | <0.001 |
| PHPT_diagnosis Yes n(%) | 1 (6.7) | 1 (100.0) | 0 (0.0) | 0.072 |
